# Machine learning in medicine: a practical introduction

**DOI:** 10.1186/s12874-019-0681-4

**Published:** 2019-03-19

**Authors:** Jenni A. M. Sidey-Gibbons, Chris J. Sidey-Gibbons

**Affiliations:** 1 Department of Engineering, University of Cambridge, Trumpington Street, Cambridge, CB2 1PZ UK; 2 Department of Surgery, Harvard Medical School, 25 Shattuck Street, Boston, 01225 Massachusetts USA; 3 Department of Surgery, Brigham and Women’s Hospital, 75 Francis Street, Boston, 01225 Massachusetts USA; 4 University of Cambridge Psychometrics Centre, Trumpington Street, Cambridge, CB2 1AG UK

**Keywords:** Medical informatics, Classification, Supervised machine learning, Programming languages, Diagnosis, Computer-assisted, Decision making, Computer-assisted

## Abstract

**Background:**

Following visible successes on a wide range of predictive tasks, machine learning techniques are attracting substantial interest from medical researchers and clinicians. We address the need for capacity development in this area by providing a conceptual introduction to machine learning alongside a practical guide to developing and evaluating predictive algorithms using freely-available open source software and public domain data.

**Methods:**

We demonstrate the use of machine learning techniques by developing three predictive models for cancer diagnosis using descriptions of nuclei sampled from breast masses. These algorithms include regularized General Linear Model regression (GLMs), Support Vector Machines (SVMs) with a radial basis function kernel, and single-layer Artificial Neural Networks. The publicly-available dataset describing the breast mass samples (*N*=683) was randomly split into evaluation (*n*=456) and validation (*n*=227) samples.

We trained algorithms on data from the evaluation sample before they were used to predict the diagnostic outcome in the validation dataset. We compared the predictions made on the validation datasets with the real-world diagnostic decisions to calculate the accuracy, sensitivity, and specificity of the three models. We explored the use of averaging and voting ensembles to improve predictive performance. We provide a step-by-step guide to developing algorithms using the open-source R statistical programming environment.

**Results:**

The trained algorithms were able to classify cell nuclei with high accuracy (.94 -.96), sensitivity (.97 -.99), and specificity (.85 -.94). Maximum accuracy (.96) and area under the curve (.97) was achieved using the SVM algorithm. Prediction performance increased marginally (accuracy =.97, sensitivity =.99, specificity =.95) when algorithms were arranged into a voting ensemble.

**Conclusions:**

We use a straightforward example to demonstrate the theory and practice of machine learning for clinicians and medical researchers. The principals which we demonstrate here can be readily applied to other complex tasks including natural language processing and image recognition.

**Electronic supplementary material:**

The online version of this article (10.1186/s12874-019-0681-4) contains supplementary material, which is available to authorized users.

## Background

Driven by an increase in computational power, storage, memory, and the generation of staggering volumes of data, computers are being used to perform a wide-range of complex tasks with impressive accuracy. Machine learning (ML) is the name given to both the academic discipline and collection of techniques which allow computers to undertake complex tasks. As an academic discipline, ML comprises elements of mathematics, statistics, and computer science. Machine learning is the engine which is helping to drive advances in the development of artificial intelligence. It is impressively employed in both academia and industry to drive the development of ‘intelligent products’ with the ability to make accurate predictions using diverse sources of data [1]. To date, the key beneficiaries of the 21 ^st^ century explosion in the availability of big data, ML, and data science have been industries which were able to collect these data and hire the necessary staff to transform their products. The learning methods developed in and for these industries offer tremendous potential to enhance medical research and clinical care, especially as providers increasingly employ electronic health records.

Two areas which may benefit from the application of ML techniques in the medical field are diagnosis and outcome prediction. This includes a possibility for the identification of high risk for medical emergencies such as relapse or transition into another disease state. ML algorithms have recently been successfully employed to classify skin cancer using images with comparable accuracy to a trained dermatologist [2] and to predict the progression from pre-diabetes to type 2 diabetes using routinely-collected electronic health record data [3].

Machine learning will is increasingly employed in combination with Natural Language Processing (NLP) to make sense of unstructured text data. By combining ML with NLP techniques, researchers have been able to derive new insights from comments from clinical incident reports [4], social media activity [5, 6], doctor performance feedback [7], and patient reports after successful cancer treatments [8]. Automatically generated information from unstructured data could be exceptionally useful not only in order to gain insight into quality, safety, and performance, but also for early diagnosis. Recently, an automated analysis of free-speech collected during in-person interviews resulted in the ability to predict transition to psychosis with perfect accuracy in a group of high-risk youths [9].

Machine learning will also play a fundamental role in the development of learning healthcare systems. Learning healthcare systems describe environments which align science, informatics, incentives, and culture for continuous improvement and innovation. In a practical sense, these systems; which could occur on any scale from small group practices to large national providers, will combine diverse data sources with complex ML algorithms. The result will be a continuous source of data-driven insights to optimise biomedical research, public health, and health care quality improvement [10].

### Machine learning

Machine learning techniques are based on algorithms – sets of mathematical procedures which describe the relationships between variables. This paper will explain the process of developing (known as *training*) and validating an algorithm to predict the malignancy of a sample of breast tissue based on its characteristics. Though algorithms work in different ways depending on their type there are notable commonalities in the way in which they are developed. Though the complexities of ML algorithms may appear esoteric, they often bear more than a subtle resemblance to conventional statistical analyses.

Given the commonalities shared between statistical and ML techniques, the boundary between the two may seem fuzzy or ill-defined. One way to delineate these bodies of approaches is to consider their primary goals. The goal of statistical methods is *inference*; to reach conclusions about populations or derive scientific insights from data which are collected from a representative sample of that population. Though many statistical techniques, such as linear and logistic regression, are capable of creating predictions about new data, the motivator of their use as a statistical methodology is to make inferences about relationships between variables. For example, if we were to create a model which described the relationship between clinical variables and mortality following organ transplant surgery for example, we would need to have insight into the factors which distinguish low mortality risk from high if we were to develop interventions to improve outcomes and reduce mortality in the future. In statistical inference, therefore, the goal is to *understand* the relationships between variables.

Conversely, in the field of ML, the primary concern is an accurate *prediction*; the ‘what’ rather than the ‘how’. For example, in image recognition, the relationship between the individual features (pixels) and the outcome is of little relevance if the prediction is accurate. This is a critical facet of ML techniques as the relationship between many inputs, such as pixels in image or video and geo-location, are complex and usually non-linear. It is exceptionally difficult to describe in a coherent way the relationships between predictors and outcomes both when the relationships are non-linear and when there are a large number of predictors, each of which make a small individual contribution to the model.

**Fig. 1:**
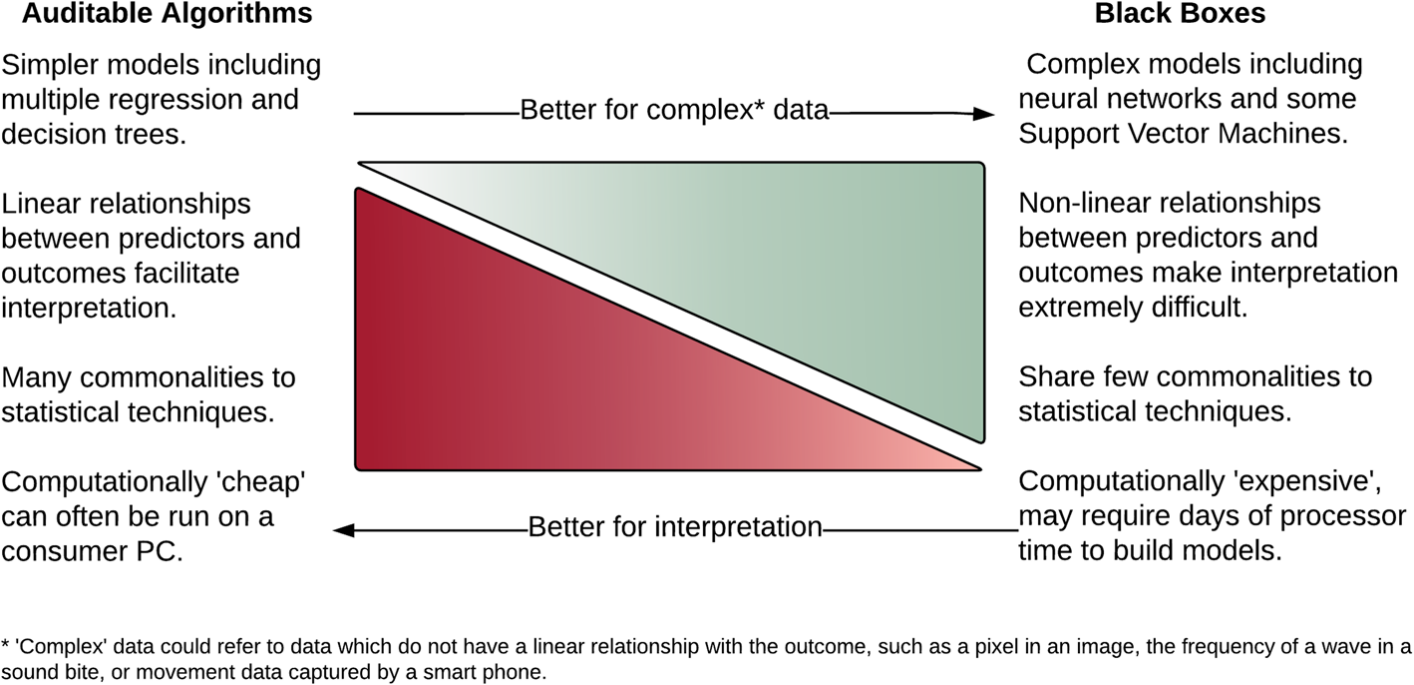
The complexity/interpretability trade-off in machine learning tools

Fortunately for the medical field, many relationships of interest are reasonably straightforward, such as those between body mass index and diabetes risk or tobacco use a lung cancer. Because of this, their interaction can often be reasonably well explained using relatively simple models. In many popular applications of ML, such a optimizing navigation, translating documents, and identifying objects in videos, understanding the relationship between features and outcomes is of less importance. This allows the use of complex non-linear algorithms. Given this key difference, it might be useful for researchers to consider that algorithms exist on a continuum between those algorithms which are easily interpretable (i.e., Auditable Algorithms) and those which are not (i.e., Black Boxes), presented visually in Fig. 1.

Interesting questions remain as to when a conventionally statistical technique becomes a ML technique. In this work, we will introduce some that computational enhancements to traditional statistical techniques, such as elastic net regression, make these algorithms performed well with big data. However, a fuller discussion of the similarities and differences between ML and conventional statistics is beyond the purview of the current paper. Interested readers are directed to materials which develop the ideas discussed here [11]. It should also be acknowledged that whilst the ’Black Box’ concept does generally apply to models which utilize non-linear transformations, such as the neural networks, work is being carried out to facilitate feature identification in complex algorithms [12].

The majority of ML methods can be categorised into two types learning techniques: those which are supervised and those which are unsupervised. Both are introduced in the following sections.

#### Supervised learning

Supervised ML refers to techniques in which a model is *trained* on a range of inputs (or features) which are associated with a known outcome. In medicine, this might represent training a model to relate a person’s characteristics (e.g., height, weight, smoking status) to a certain outcome (onset of diabetes within five years, for example). Once the algorithm is successfully trained, it will be capable of making outcome predictions when applied to new data. Predictions which are made by models trained using supervised learning can be either discrete (e.g., positive or negative, benign or malignant) or continuous (e.g., a score from 0 to 100).

A model which produces discrete categories (sometimes referred to as classes) is referred to as a *classification* algorithm. Examples of classification algorithms include those which, predict if a tumour is benign or malignant, or to establish whether comments written by a patient convey a positive or negative sentiment [2, 6, 13]. In practice, classification algorithms return the probability of a class (between 0 for impossible and 1 for definite). Typically, we would transform any probability greater than.50 into a class of 1, but this threshold may be altered to improve algorithm performance as required. This paper provides an example of a classification algorithm in which a diagnosis is predicted.

A model which returns a prediction of a continuous value is known as a *regression* algorithm. The use of the term regression in ML varies from its use in statistics, where regression is often used to refer to both *binary outcomes* (i.e., logistic regression) and continuous outcomes (i.e., linear regression). In ML, an algorithm which is referred to as a regression algorithm might be used to predict an individual’s life expectancy or tolerable dose of chemotherapy.

Supervised ML algorithms are typically developed using a dataset which contains a number of variables and a relevant outcome. For some tasks, such as image recognition or language processing, the variables (which would be pixels or words) must be processed by a feature selector. A feature selector picks identifiable characteristics from the dataset which then can be represented in a numerical matrix and understood by the algorithm. In the examples above, a feature may be the colour of a pixel in an image or the number of times that a word appears in a given text. Using the same examples, outcomes may be whether an image shows a malignant or benign tumour or whether transcribed interview responses indicate predisposition to a mental health condition.

Once a dataset has been organised into features and outcomes, a ML algorithm may be applied to it. The algorithm is iteratively improved to reduce the error of prediction using an optimization technique.

**Fig. 2:**
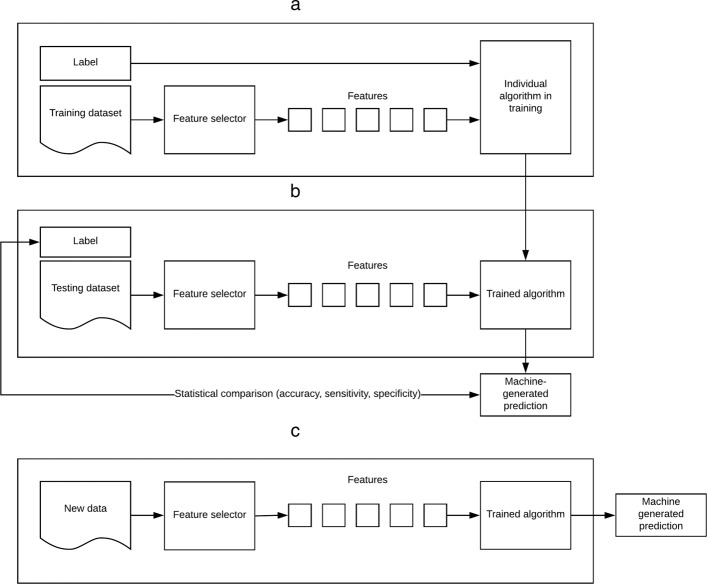
Overview of supervised learning. **a** Training **b** Validation **c** Application of algorithm to new data

Note that, when training ML algorithms, it is possible to over-fit the algorithm to the nuances of a specific dataset, resulting in a prediction model that does not generalise well to new data. The risk of over-fitting can be mitigated using various techniques. Perhaps the most straight-forward approach, which will be employed in this work, is to split our dataset into two segments; a training segment and a testing segment to ensure that the trained model can generalize to predictions beyond the training sample. Each segment contains a randomly-selected proportion of the features and their related outcomes. This allows the algorithm to associate certain features, or characteristics, with a specific outcome, and is known as *training* the algorithm. Once training is completed, the algorithm is applied to the features in the testing dataset without their associated outcomes. The predictions made by the algorithm are then compared to the known outcomes of the testing dataset to establish model performance. This is a necessary step to increase the likelihood that the algorithm will generalise well to new data. This process is illustrated graphically in Fig. 2.

#### Unsupervised Machine Learning

**Fig. 3:**
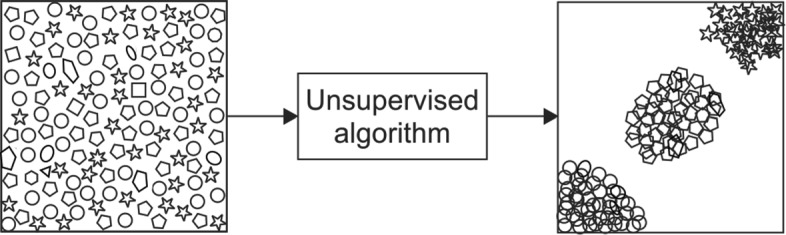
A visual illustration of an unsupervised dimension reduction technique

In contrast with supervised learning, unsupervised learning does not involve a predefined outcome. In unsupervised learning, patterns are sought by algorithms without any input from the user. Unsupervised techniques are thus exploratory and used to find undefined patterns or clusters which occur within datasets. These techniques are often referred to as *dimension reduction techniques* and include processes such as principal component analysis, latent Dirichlet analysis and t-Distributed Stochastic Neighbour Embedding (t-SNE) [14–16]. Unsupervised learning techniques are not discussed at length in this work, which focusses primarily on supervised ML. However, unsupervised methods are sometimes employed in conjunction with the methods used in this paper to reduce the number of features in an analysis, and are thereby worth mention. By compressing the information in a dataset into fewer features, or dimensions, issues including multiple-collinearity or high computational cost may be avoided. A visual illustration of an unsupervised dimension reduction technique is given in Fig. 3. In this figure, the raw data (represented by various shapes in the left panel) are presented to the algorithm which then groups the data into clusters of similar data points (represented in the right panel). Note that data which do not have sufficient commonality to the clustered data are typically excluded, thereby reducing the number of features within of the dataset.

In a similar way to the supervised learning algorithms described earlier, also share many similarities to statistical techniques which will be familiar to medical researchers. Unsupervised learning techniques make use of similar algorithms used for clustering and dimension reduction in traditional statistics. Those familiar with Principal Component Analysis and factor analysis will already be familiar with many of the techniques used in unsupervised learning.

### What this paper will achieve

This paper provides a pragmatic example using supervised ML techniques to derive classifications from a dataset containing multiple inputs. The first algorithm we introduce, the regularized logistic regression, is very closely related to multivariate logistic regression. It is distinguished primarily by the use of a regularization function which both reduces the number of features in the model and attenuates the magnitude of their coefficients. Regularization is, therefore, suitable for datasets which contain many variables and missing data (known as *high sparsity datasets*), such as the term-document matrices which are used to represent text in text mining studies.

The second algorithm, a Support Vector Machine (SVM), gained popularity among the ML community for its high performance deriving accurate predictions in situations where the relationship between features and the outcome is non-linear. It uses a mathematical transformation known as the *kernel trick*, which we describe in more detail below.

Finally, we introduce an Artificial Neural Network (ANN), in which complex architecture and heavily modifiable parameters have led to it’s widespread use in many challenging applications, including image and video recognition. The addition of speciality neural networks, such as recurrent or convolutional networks, to ANNs has resulted in impressive performance on a range of tasks. Being highly parametrized models, ANNs are prone to over-fitting. Their performance may be improved using a regularization technique, such as DropConnect.

The ultimate goal of this manuscript is to imbue clinicians and medical researchers with both a foundational understanding of what ML is, how it may be used, as well as the practical skills to develop, evaluate, and compare their own algorithms to solve prediction problems in medicine.

### How to follow this paper

We provide a conceptual introduction alongside practical instructions using code written for the R Statistical Programming Environment, which may be easily modified and applied to other classification or regression tasks. This code will act as a framework upon which researchers can develop their own ML studies. The models presented here may be fitted to diverse types of data and are, with minor modifications, suitable for analysing text and images.

This paper is divided into sections which describe the typical stages of a ML analysis: preparing data, training algorithms, validating algorithms, assessing algorithm performance, and applying new data to the trained models.

Throughout the paper, examples of R code used to the run the analyses are presented. The code is given in full in Additional file 1. The data which was used for these analyses are available in Addition file 2.

## Methods

**Fig. 4:**
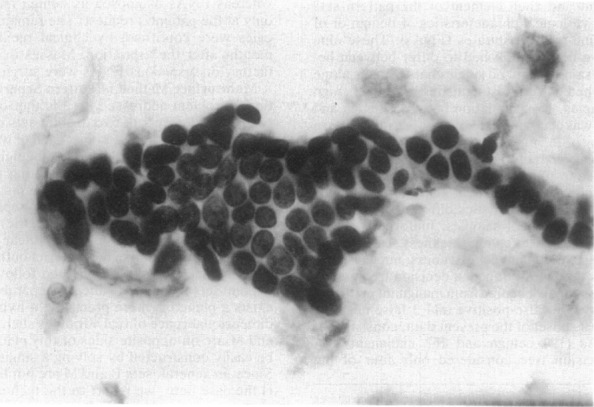
An example of an image of a breast mass from which dataset features were extracted

The dataset used in this work is the Breast Cancer Wisconsin Diagnostic Data Set. This dataset is publicly available from the University of California Irvine (UCI) Machine Learning Repository [17]. It consists of characteristics, or features, of cell nuclei taken from breast masses which were sampled using fine-needle aspiration (FNA), a common diagnostic procedure in oncology. The clinical samples used to form this dataset were collected from January 1989 to November 1991. Relevant features from digitised images of the FNA samples were extracted through the methods described in Refs. [13, 18, 19]. An example of one of the digitised images from an FNA sample is given in Fig. 4.

A total of 699 samples were used to create this dataset. This number will be referred to as the *number of instances*. Each instance has an I.D. number, diagnosis, and set of features attributed to it. While the *Sample I.D.* is unique to that instance, the diagnosis, listed as *class* in the dataset, can either be malignant or benign, depending if the FNA was found to be cancerous or not. In this dataset, 241 instances were diagnosed as malignant, and 458 instances were found to be benign. Malignant cases have a class of four, and benign cases have a class of two. This class, or diagnosis, is the *outcome* of the instance.

The features of the dataset are characteristics identified or calculated from each FNA image. There are nine features in this dataset, and each is valued on a scale of 1 to 10 for a particular instance, 1 being the closest to benign and 10 being the most malignant [18]. Features range from descriptors of cell characteristics, such as *Uniformity of Cell Size* and *Uniformity of Cell Shape*, to more complex cytological characteristics such as *Clump Thickness* and *Marginal Adhesion*. All nine features, along with the Instance No., Sample I.D., and Class are listed in Table 1. The full dataset is a matrix of 699 × 12 (one identification number, nine features, and one outcome per instance).

**Table 1:** Attributes of the dataset

		Features									Outcome
Instance	Sample		Cell	Cell		Epithelial	Bare	Bland	Normal		Class
No.	I.D.	Thickness	Size	Shape	Adhesion	Size	Nuclei	Chromatin	Nucleoli	Mitoses	(Diagnosis)
1	1000025	5	1	1	1	2	1	3	1	1	2
2	1002945	5	4	4	5	7	10	3	2	1	2
3	1015425	3	1	1	1	2	2	3	1	1	2
.											
.											
.											
699	897471	4	8	8	5	4	5	10	4	1	4

This dataset is simple and therefore computationally efficient. The relatively low number of features and instances means that the analysis provided in this paper can be conducted using most modern PCs without long computing times. Although the principals are the same as those described throughout the rest of this paper, using large datasets to train Machine learning algorithms can be computationally intensive and, in some cases, require many days to complete. The principals illustrated here apply to datasets of any size.

## Using R

The R Statistical Programming Language is an open-source tool for statistics and programming which was developed as an extension of the S language. R is supported by a large community of active users and hosts several excellent packages for ML which are both flexible and easy to use. R is a computationally efficient language which is readily comprehensible without special training in computer science. The R language is similar to many other statistical programming languages, including MATLAB, SAS, and STATA. Packages for R are arranged into different task views on the Comprehensive R Archive Network. The Machine Learning and Statistical Learning task view currently lists almost 100 packages dedicated to ML.

Many, if not most, R users access the R environment using RStudio, an open-source integrated developer environment (IDE) which is designed to make working in R more straightforward. We recommend that readers of the current paper download the latest version of both R and RStudio and access the environment through the RStudio application. Both R and RStudio are free to use and available for use under an open-source license.

## Conducting a machine learning analysis

The following section will take you through the necessary steps of a ML analysis using the Wisconsin Cancer dataset.

1. Importing and preparing the dataset.
2. Training the ML algorithms.
3. Testing the ML algorithms.
4. Assessing sensitivity, specificity and accuracy of the algorithms.
5. Plotting receiver operating characteristic curves.
6. Applying new data to the trained models.

### 1. Importing and preparing the dataset.

**Fig. 5:**
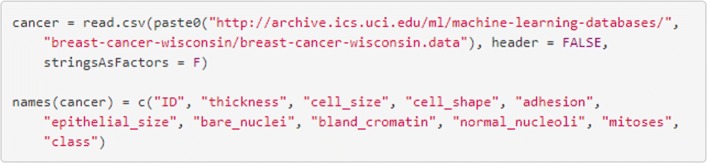
Import the data and label the columns

The dataset can be downloaded directly from the UCI repository using the code in Fig. 5.

**Fig. 6:**
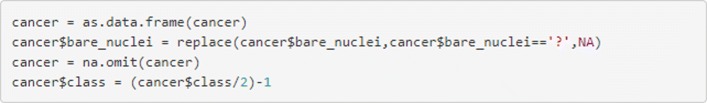
Remove missing items and restore the outcome data

We first modify the data by re-scoring missing data from ‘?’ to NA, removing any rows with missing data and re-scoring the class variables from 2 and 4 to 0 and 1, where 0 indicates the tumour was benign and 1 indicates that it was malignant. Recall that a dataset with many missing data points is referred to as a *sparse* dataset. In this dataset there are small number of cases (n =16) with at least one missing value. To simplify the analytical steps, we will remove these cases, using the code in Fig. 6.

Datasets used for supervised ML are most easily represented in a matrix similar to the way Table 1 is presented. The *n* columns are populated with the *n*−1 features, with the single remaining column containing the outcome. Each row contains an individual instance. The features which make up the training dataset may also be described as *inputs* or *variables* and are denoted in code as *x*. The outcomes may be referred to as the *label* or the *class* and are denoted using *y*.

**Fig. 7:**
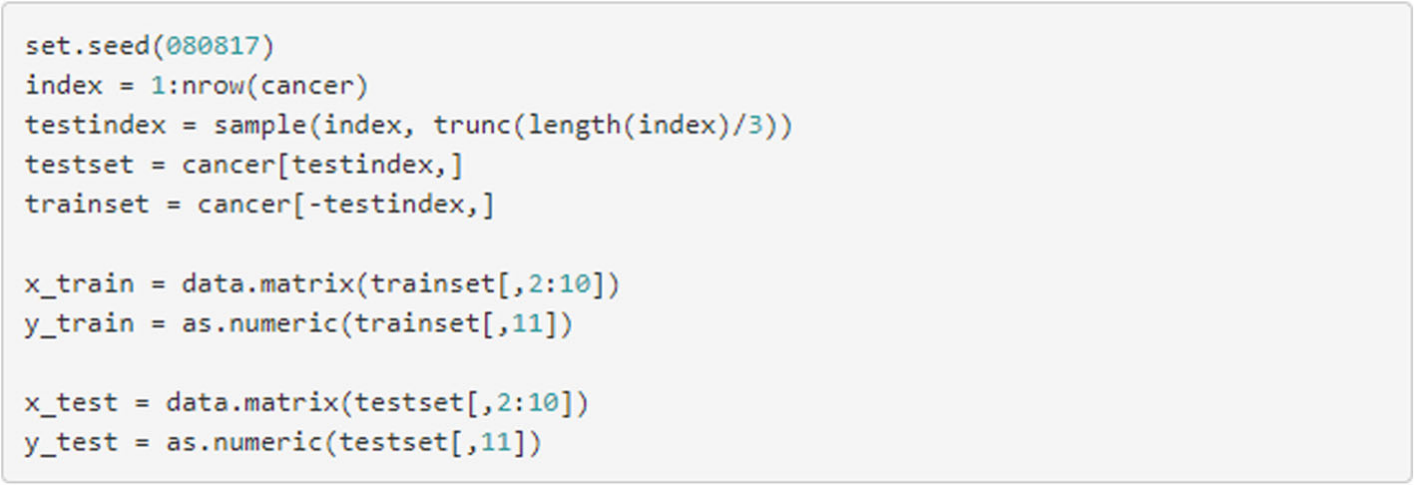
Split the data into training and testing datasets

Recall that it is necessary to train a supervised algorithm on a training dataset in order to ensure it generalises well to new data. The code in Fig. 7 will divide the dataset into two required segments, one which contains 67% of the dataset, to be used for training; and the other, to be used for evaluation, which contains the remaining 33%.

### 2. Training the ML algorithms

Now that we have arranged our dataset into a suitable format, we may begin training our algorithms. These ML algorithms which we will use are listed below and detailed in the following section.

- Logistic regression using Generalised Linear Models (GLMs) with $\mathscr {L}_{1}$ Least Absolute Selection and Shrinkage Operator (LASSO) regularisation.
- Support Vector Machines (SVMs) with a radial basis function (RBF) kernel.
- Artificial Neural Networks (ANNs) with a single hidden layer.

### Regularised regression using Generalised Linear Models (GLMs)

Regularised General Linear Models (GLMs) have demonstrated excellent performance in some complex learning problems, including predicting individual traits from on-line digital footprints [20], classifying open-text reports of doctors’ performance [7], and identifying prostate cancer by desorption electro-spray ionization mass spectrometric imaging of small metabolites and lipids [21].

When fitting GLMs using datasets which have a large number of features and substantial sparsity, model performance may be increased when the contribution of each of the included features to the model is reduced (or penalised) using regularisation, a process which also reduces the risk of over-fitting. Regularisation effectively reduces both the number of coefficients in the model and their magnitudes, making especially it suitable for big datasets that may have more features than instances. In this example, feature selection is guided by the Least Absolute Shrinkage and Selection Operator (LASSO). Other forms of regularisation are available, including Ridge Regression and the Elastic Net (which is a linear blend of both Ridge and LASSO regularisation) [22]. An accessible, up-to-date summary of LASSO and other regularisation techniques is given in Ref [23].

Regularised GLMs are operationalised in R using the **glmnet** package [24]. The code below demonstrates how the GLM algorithm is fitted to the training dataset. In the **glmnet** package, the regularistion parameter is chosen using the numerical value referred to as alpha. In this package, a alpha value of 1 selects LASSO regularisation where as alpha 0 selects Ridge regularization, a value between between 0 and 1 selects a linear blend of the two techniques known as the Elastic Net [22].

**Fig. 8:**
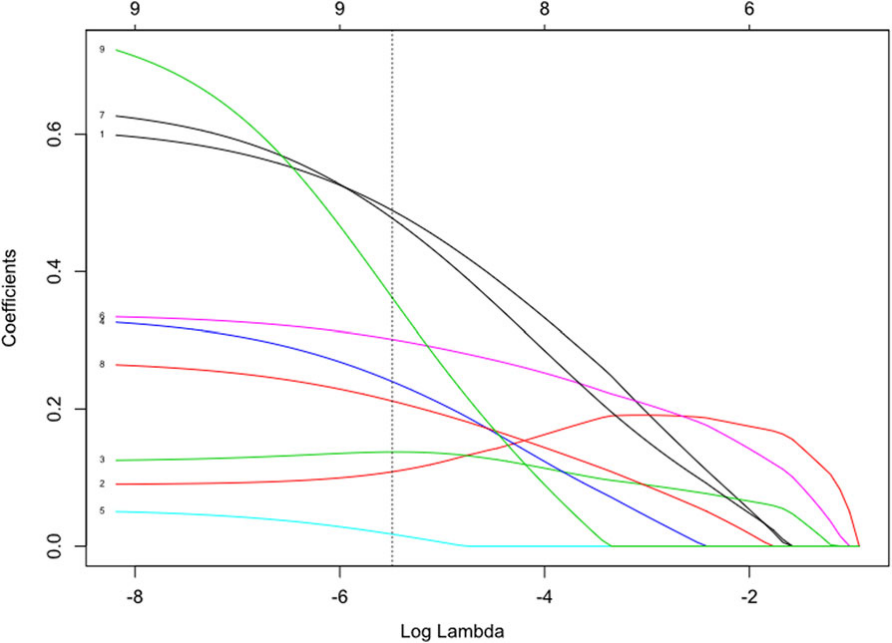
Regression coefficients for the GLM model. The figure shows the coefficients for the 9 model features for different values of log(*λ*). log(*λ*) values are given on the lower x-axis and number of features in the model are displayed above the figure. As the size of log(*λ*) decreases the number of variables in the model (i.e. those with a nonzero coefficient) increases as does the magnitude of each feature. The vertical dotted line indicates the value of log(*λ*) at which the accuracy of the predictions is maximized

**Fig. 9:**
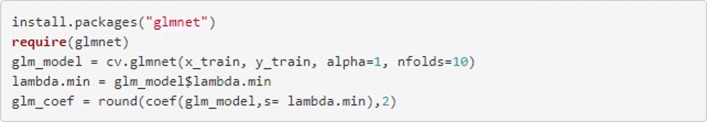
Fit the GLM model to the data and extract the coefficients and minimum value of lambda

nFold cross-validation is used to ascertain the optimal value of lambda (*λ*), the regularisation parameter. The value of (*λ*) which minimizes prediction error is stored in the glm_model$lambda.min object. The smaller the *λ* value, the greater the effect of regularisation upon the number of features in the model and their respective coefficients. Figure 8 shows the effect of different levels of log(*λ*). The optimal value of log(*λ*) is indicated using the vertical broken line (shown here at x = -5.75). The rightmost dotted line indicates the most parsimonious value of log(*λ*) which is within 1 standard deviation of the absolute minimum value. Note that the random nature of cross-validation means that values of log(*λ*) may differ slightly between analyses. The integers are given above Fig. 8 (0-9) relate to the number of features included in the model. The code shown in Fig. 9 fits the GLM algorithm to the data and extracts the minimum value of *λ* and the weights of the coefficients.

**Fig. 10:**
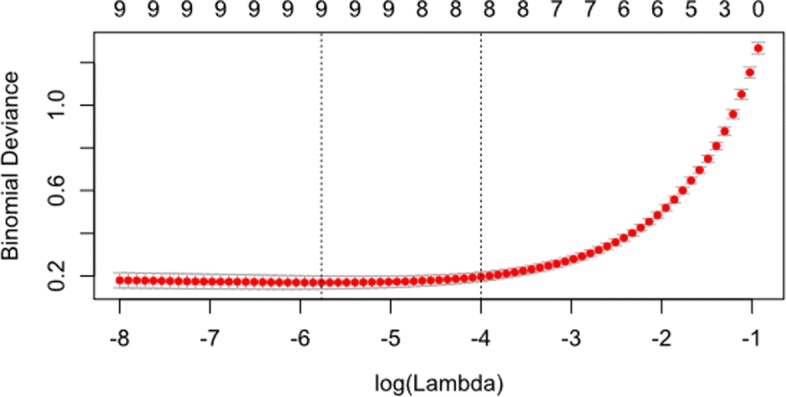
Cross-validation curves for the GLM model. The figure shows the cross-validation curves as the red dots with upper and lower standard deviation shown as error bars

**Fig. 11:**
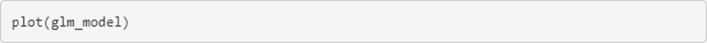
Plot the cross-validation curves for the GLM algorithm

Figure 10 shows the cross-validation curves for different levels of log(*λ*). This figure can be plotted using the code in Fig. 11.

**Fig. 12:**
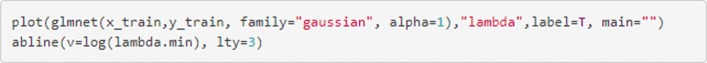
Plot the coefficients and their magnitudes

Figure 8 shows magnitude of the coefficients for each of the variables within the model for different values of log(*λ*). The vertical dotted line indicates the value of log(*λ*) which minimises the mean squared error established during cross-validation. This figure can be augmented with a dotted vertical line indicating the value of log(*λ*) using the abline() function, shown in Fig. 12.

### Support Vector Machines (SVMs)

Support Vector Machine (SVM) classifiers operate by separating the two classes using a linear decision boundary called the hyperplane. The hyperplane is placed at a location that maximises the distance between the hyperplane and instances [25].

**Fig. 13:**
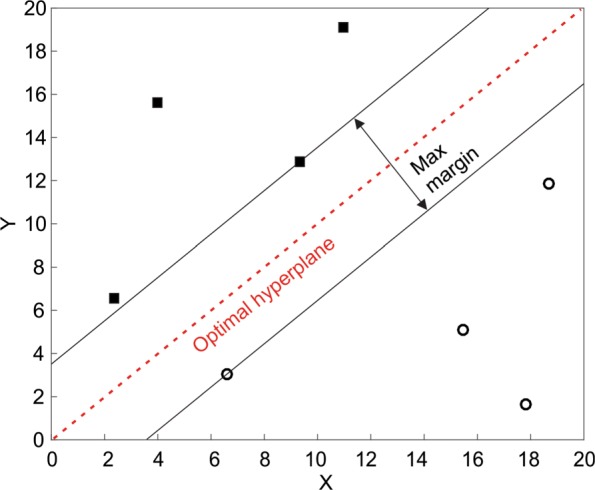
A SVM Hyperplane The hyperplane maximises the width of the decision boundary between the two classes

**Fig. 14:**
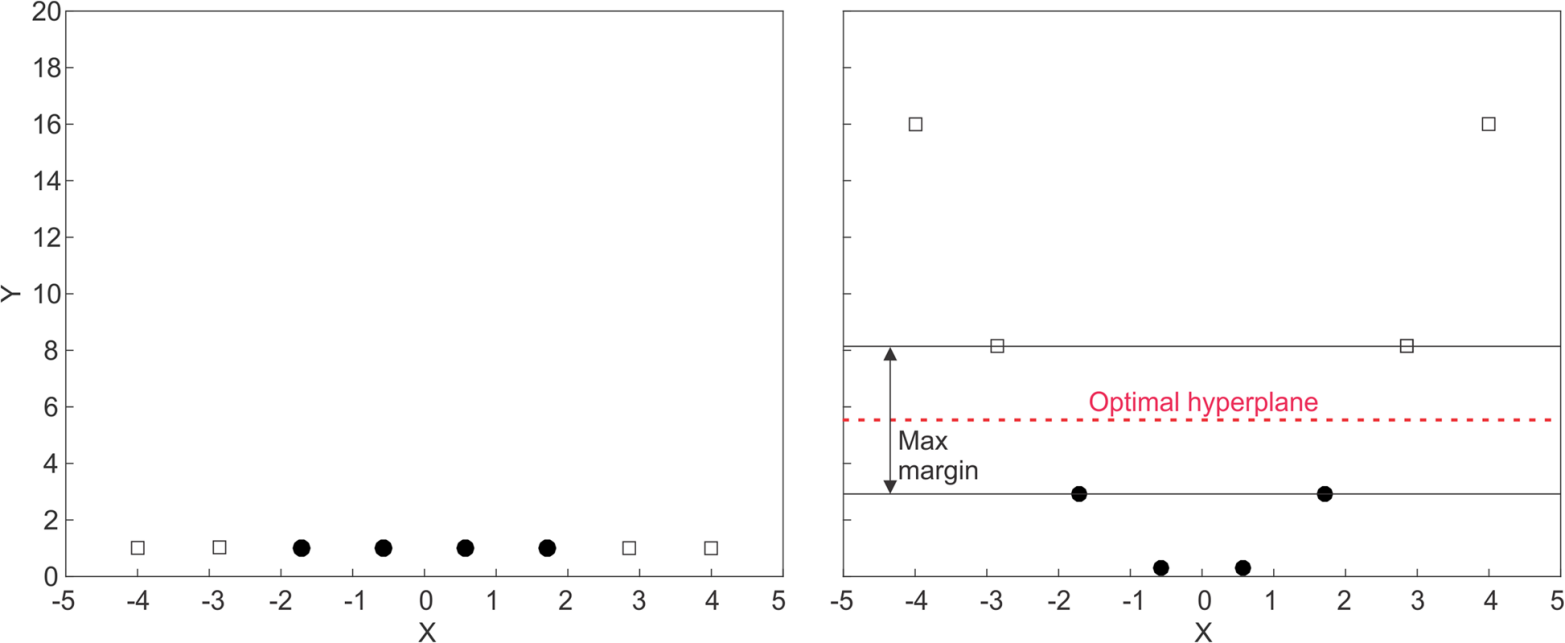
The kernel trick The kernel trick modifies the feature space allowing separation of the classes with a linear hyperplane

Fig. 13 depicts an example of a linear hyperplane that perfectly separates between two classes. In real-world examples, it may not be possible to adequately separate the two classes using a linear hyperplane. By maximising the width of the decision boundary then the generalisability of the model to new data is optimised. Rather than employ a non-linear separator such as a high-order polynomial, SVM techniques use a method to transform the feature space such that the classes do become linearly separable. This technique, known as the kernel trick, is demonstrated in Fig. 14.

Fig. 14 shows an example of a two classes that are not separable using a linear separator. By projecting the data to *X*^2^, they become linearly separable using the *y*=5 hyperplane. A popular method for kernel transformation in high-dimensional space is the radial basis function (RBF).

**Fig. 15:**
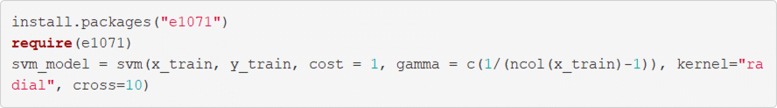
Fit the SVM algorithm to the data

The SVM algorithm is fitted to the data using a function, given in Fig. 15, which is arranged in a similar way to the regularised regression shown above.

Further exploration of SVM which attempt to fit separating hyperplanes following different feature space transformations is possible by altering the kernel argument to “linear”, “radial”, “polynomial”, or “sigmoid”.

### Artificial Neural Networks (ANNs)

Artificial Neural Networks (ANNs) are algorithms which are loosely modelled on the neuronal structure observed in the mammalian cortex. Neural networks are arranged with a number of input neurons, which represent the information taken from each of the features in the dataset. which feed into any number of hidden layers before passing to an output layer in which the final decision is presented. As information passes through the ’neurons’, or nodes, where is is multiplied by the weight of the neuron (plus a constant bias term) and transformed by an activation function. The activation function applies a non-linear transformation using a simple equation shown in Eq. 1.

In recurrent ANNs, a process is undertaken in which the prediction errors are fed back through the network before modifying the weights of each neural connection is altered until error level is minimised, a process known as backpropagation [26].

1 $$ y = activation(\Sigma(weight\times input)+bias)   $$

Deep Neural Networks (DNNs) refers to neural networks which have many hidden layers. Deep learning, which may utilise DNNs, has produced impressive results when employed in complex tasks using very high dimensional data, such as image recognition [27] and computer-assisted diagnosis of melanoma [2].

DNNs are heavily parametrised and, resultantly, can be prone to over-fitting models to data. Regularisation can, like the GLM algorithm described above, be used prevent this. Other strategies to improve performance can include dropout regularisation, where some number of randomly-selected units are omitted from the hidden layers during training [28].

**Fig. 16:**
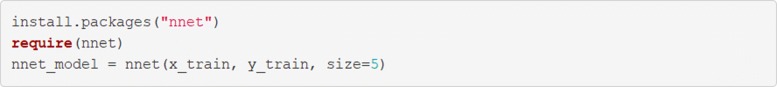
Fit the ANN algorithm to the data

The code in Fig. 16 demonstrates the code for fitting a neural network. This is straightforward, requiring the x and y datasets to be defined, as well as the number of units in the hidden layer using the size argument.

### 3. Testing the ML algorithms

**Fig. 17:**
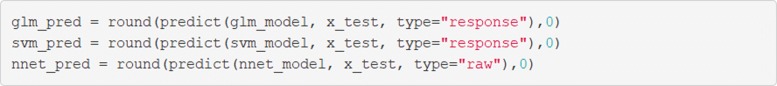
Extract predictions from the trained models on the new data

In order to test the performance of the trained algorithms, it is necessary to compare the predictions which the algorithm has made on data other than the data upon which it was trained with the true outcomes for that data which we have known but we did not expose the algorithm to. To accomplish this in he R programming environment, we would create a vector of model predictions using the x_test matrix, which can be compared to the y_test vector to establish performance metrics. This is easily achievable using the predict() function, which is included in the stats package in the R distribution. The nnet package contains a minor modification to the predict() function, and as such the type argument is set to ‘raw’, rather than ‘response’ for the neural network. This code is given in Fig. 17.

### 4. Assessing the sensitivity, specificity and accuracy of the algorithms

Machine learning algorithms for classification are typically evaluated using simple methodologies that will be familiar to many medical researchers and clinicians. In the current study, we will use sensitivity, specificity, and accuracy to evaluate the performance of the three algorithms. *Sensitivity* is the proportion of true positives that are correctly identified by the test, *specificity* is the proportion of true negatives that are correctly identified by the test and the *accuracy* is the proportion of the times which the classifier is correct [29]. Equations used to calculate sensitivity, specificity, and accuracy are given below.

2 $$\begin{array}{*{20}l} \text{Sensitivity} =& \text{true positives} / \text{actual positives} \end{array} $$

3 $$\begin{array}{*{20}l} \text{Specificity} =& \text{true negatives} / \text{actual negatives} \end{array} $$

4 $$\begin{array}{*{20}l} \text{Accuracy} =& (\text{true positives} + \text{true negatives)}/\text{total}\\ &\text{predictions} \end{array} $$

#### Confusion matrices

Data from classifiers are often represented in a *confusion matrix* in which the classifications made by the algorithm (e.g., pred_y_svm) are compared to the true classifications (which the algorithms were blinded to) in the dataset (i.e., y_test). Once populated, the confusion matrix provides all of the information needed to calculate sensitivity, specificity, and accuracy manually. An example of an unpopulated confusion matrix is demonstrated in Table 2.

**Table 2:** Example confusion matrix

		Actual Outcome	
		Negative	Positive
Predicted	Negative	True Negative (TN)	False Negative (TN)
Out come	Positive	False Positive (FP)	True Positive (TP)

**Fig. 18:**
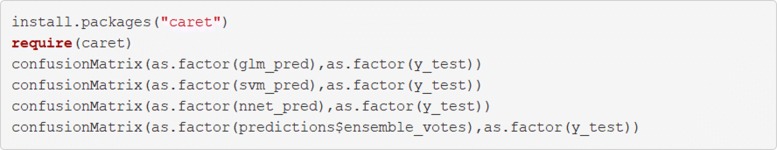
Create confusion matrices for the three algorithms

Confusion matrices can be easily created in R using the **caret** package. The confusionMatrix() function creates a confusion matrix and calculates sensitivity, specificity, and accuracy. The confusionMatrix() function requires a binary input for the predictors whereas the pred() functions used earlier produce a vector of continuous values between 0 and 1, in which a larger value reflects greater certainty that the sample was positive. Before evaluating a binary classifier, a cut-off threshold must be decided upon. The round() function used in the code shown in Fig. 18 effectively sets a threshold of >.50 for a positive prediction by rounding values ≤.50 down to 0 and values >.50 up to 1. While this is sufficient for this teaching example, users may wish to evaluate the optimal threshold for a positive prediction as this may differ from.50. The populated confusion matrix for this example is shown in Table 3 and is displayed alongside sensitivity, specificity, and accuracy.

**Table 3:** Confusion matrix shown alongside sensitivity, specificity, and accuracy for each algorithm described here

			Actual outcome				
			Benign (0)	Malignant (1)	Sensitivity	Specificity	Accuracy
Predicted Outcomes	GLM	Benign (0)	148	10	.99	.87	.95
		Malignant (1)	2	67			
	SVM	Benign (0)	146	5	.97	.94	.96
		Malignant (1)	4	72			
	ANN	Benign (0)	148	11	.99	.86	.94
		Malignant (1)	2	66			

### 5. Plotting receiver operating characteristic curves

**Fig. 19:**
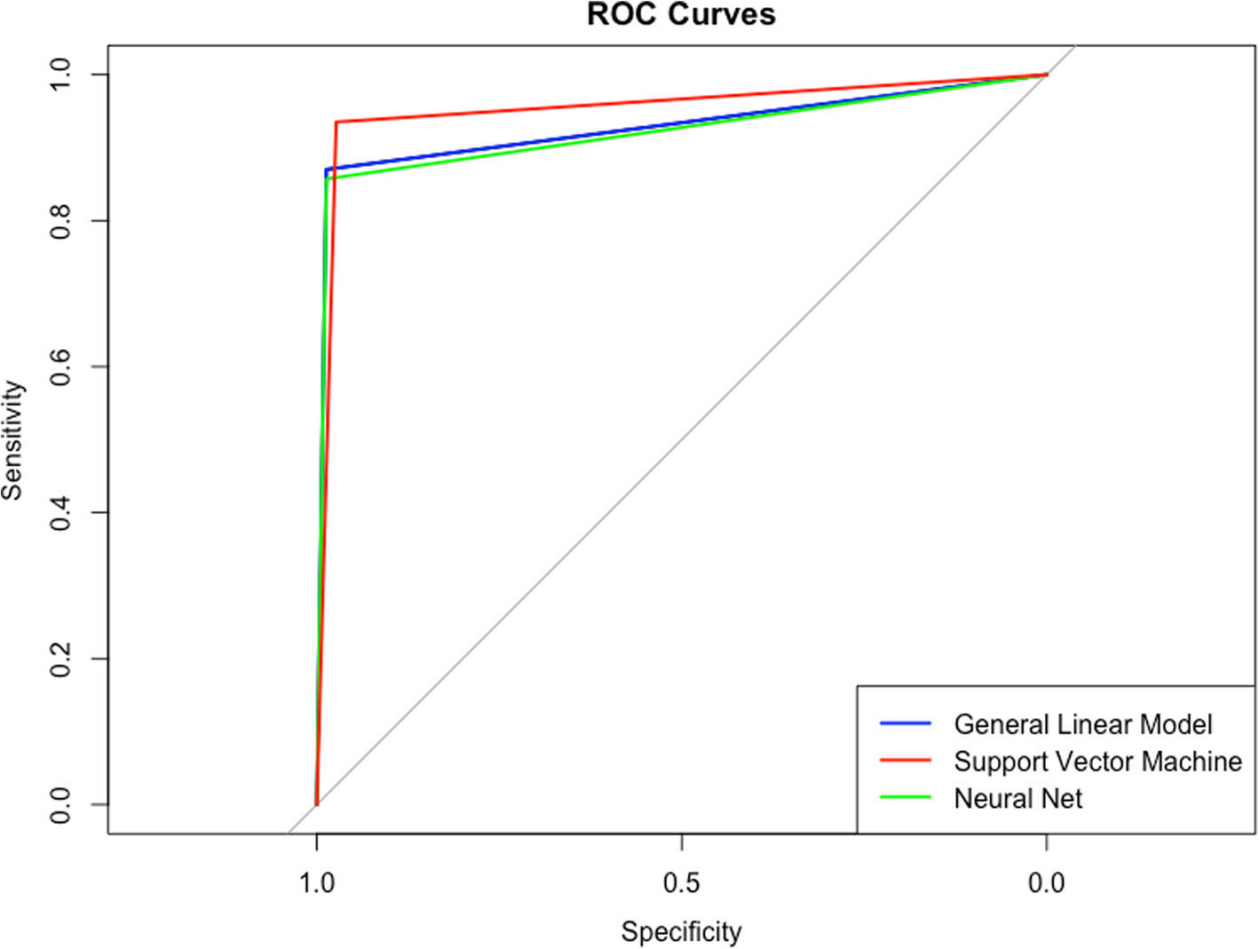
Draw received operating curves and calculate the area under them

**Fig. 20:**
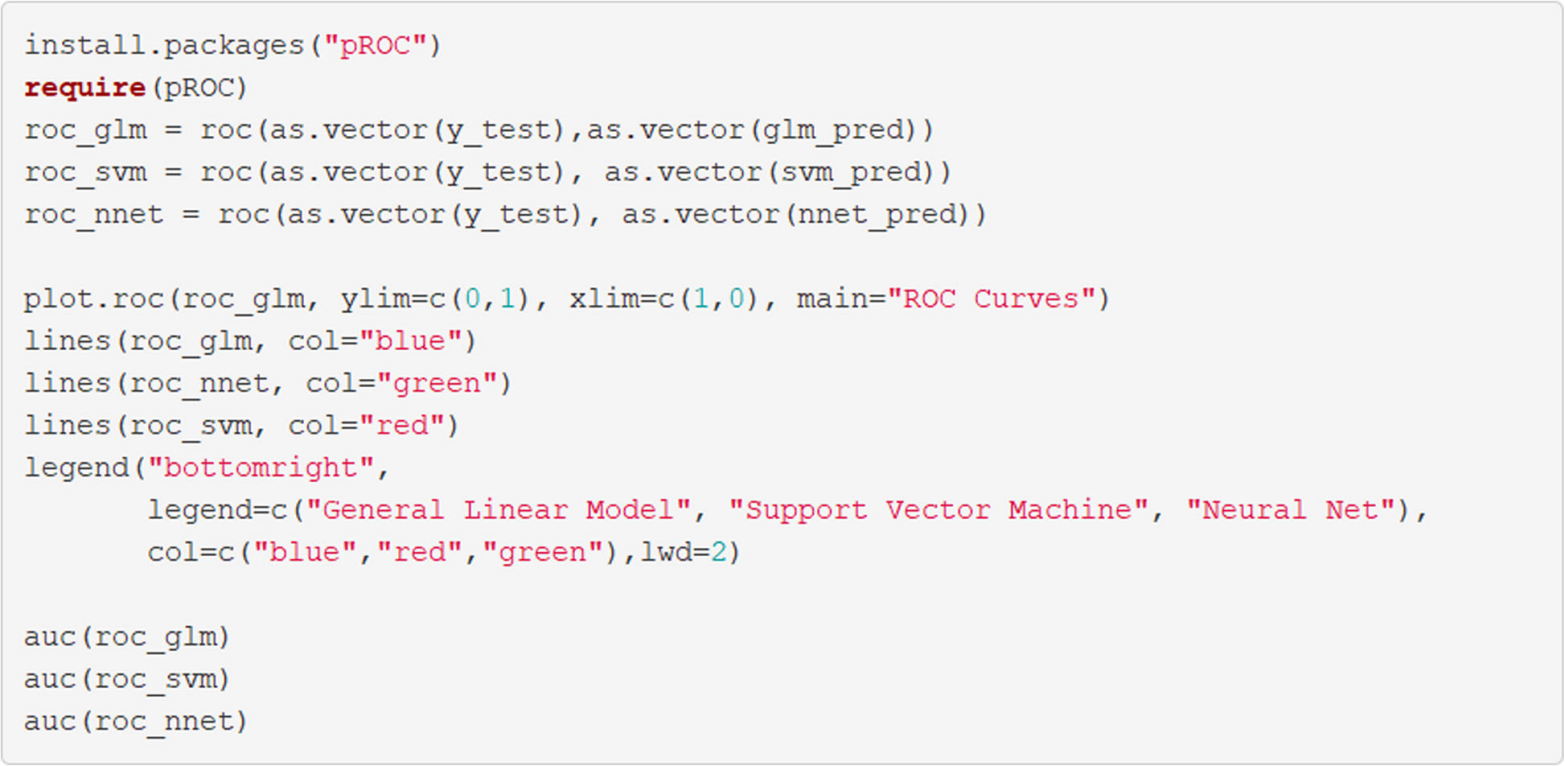
Receiver Operating Characteristics curves

Receiver operating characteristics curves are useful and are shown in the code in Fig. 19 using the **pROC** package. An example output is given in Fig. 20. These curves illustrate the relationship between the model’s sensitivity (plotted on the *y*-axis) and specificity (plotted on the *x*-axis). The grey diagonal line is reflective of as-good-as-chance performance and any curves which are plotted to the left of that line are performing better than chance. Interpretation of ROC curves is facilitated by calculating the area under each curve (AUC) [30]. The AUC gives a single value which explains the probability that a random sample would be correctly classified by each algorithm. In this example all models perform very well but the SVM algorithm shows the best performance, with AUC =.97 compared to the ANN (AUC =.95) and the LASSO-regularized regression (AUC =.94).

### 6. Applying new data to the trained models

Despite many similarities, ML is differentiated from statistical inference by its focus on predicting real-life outcomes from new data. As such, we develop models not to infer the relationships between variables but rather to produce reliable predictions from new data (though, as we have demonstrated, prediction and inference are not mutually exclusive).

**Fig. 21:**
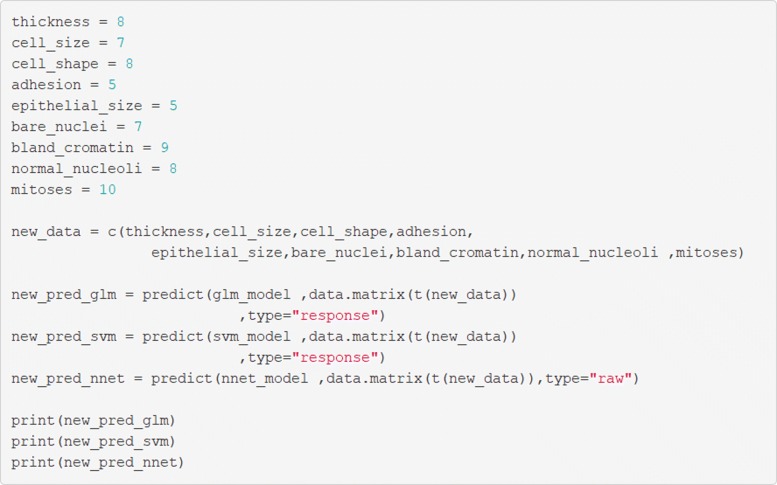
Apply new data to the trained and validated algorithm

In order to use the trained models to make predictions from data we need to construct either a vector (if there is a single new case) or a matrix (if there are multiple new cases). We need to ensure that the new data are entered into the model in the same order as the x_train and x_test matrices. In this case, we need to enter new data in the order of *thickness*, *cell size*, *cell shape*, *adhesion*, *epithelial size*, *bare nuclei*, *bland cromatin*, *normal nucleoli*, and *mitoses*. The code in Fig. 21 demonstrates how these data are represented in a manner that allows them to be processed by the trained model. Note that all three algorithms return predictions that suggest there is a near-certainty that this particular sample is malignant.

## Additional ML techniques

### Reducing prediction error; the case for ensembles.

When working to maximise the performance of a predictive model, it can be beneficial to group different algorithms together to create a more robust prediction in a process known as ensemble learning [24]. There are too many ensemble techniques to adequately summarize here, but more information can be found in Ref. [23].

**Fig. 22:**
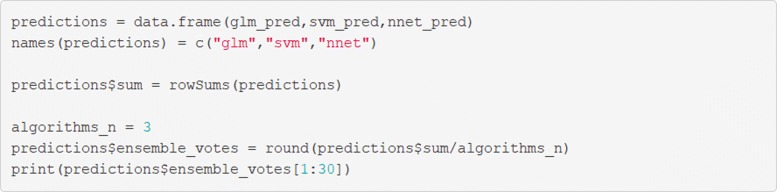
Create predictions from the ensemble

The principal of ensemble learning can be demonstrated using a un-weighted voting algorithm with R code. The code in Fig. 22 can be used to demonstrate the process of developing both an averaging and and voting algorithm.

### Natural language processing

Another common use for classification algorithms is in Natural Language Processing (NLP), the branch of ML in which computers are taught to interpret linguistic data. One popular example of NLP is in sentiment analysis, which involves ML algorithms trained to classify texts into different categories relating to the sentiment they convey; usually positive, negative, or neutral. We will give an overview of how features can be extracted from text and then used in the framework we have introduced above.

A linguistic dataset (also known as a *corpus*) comprises a number of distinct *documents*. The documents can be broken down into smaller *tokens* of text, such as the individual words contained within. These tokens can be used as the features in a ML analysis as demonstrated above. In such an analysis, we arrange the x_train matrix such that the rows represent the individual documents and the tokenized features are represented in the columns. This arrangement for linguistic analysis is known as a *term-document matrix* (TDM).

In its most basic form, each row of the TDM represents a simple count of the words which were used in a document. In this case, the width of a TDM is equal to the number of unique words in the entire corpus and, for each document, the value any given cell will either be 0 if the word does not appear in that comment or 1 if it does. Arranging a document this way leads to two issues: firstly, that the majority of the matrix likely contains null values (an issue known as *sparsity*); and secondly, that many of the documents contain the most common words in a language (e.g., “the”, “a”, or “and”) which are not very informative in analysis. Refining the TDM using a technique known as a term-frequency-inverse document frequency (TF-IDF) weighting can reduce the value of certain common words in the matrix which may be less informative and increase the value of less common words, which may be more informative. It is also possible to remove uninformative words using a pre-defined dictionary known as a *stop words* dictionary.

In a TDM, words can be tokenized individually, known as *unigrams*, or as groups of sequential words, known a nGrams where *n* is the number of words extracted in the token (*i.e, bi-gram or tri-gram extraction*). Such extraction can mitigate issues caused by grammatical nuances such as negation (e.g., “I never said she stole my money.”). Some nuances are more difficult to analyse robustly, especially those used commonly in spoken language, such as emphasis or sarcasm. For example, the sentence above about the stolen money could have at least 7 different meanings depending on where the emphasis was placed.

A TDM can be easily developed in R using the tools provided in the tm package. In Table 4, we demonstrate a simple uniGram (single word) TDM without TF-IDF weighting.

**Table 4:** Example of an unweighted term-document matrix (TDM) with outcomes

		Features								Outcome
documentID	service	a	bad	doctor	great	kind	satisfied	service	unhappy	Class
1	001312	1	0	0	1	0	1	1	0	1
2	001345	1	1	0	0	0	0	1	1	0
3	001312	0	0	1	1	0	0	0	0	1
.										
.										
.										
699	001345	1	1	0	0	0	1	0	0	0

**Fig. 23:**
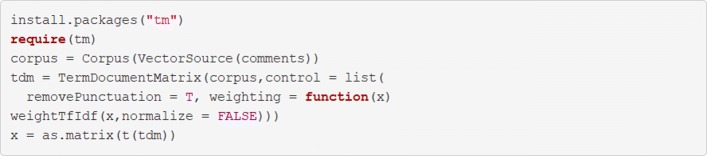
Create a term document matrix

The code in Fig. 23 demonstrates the process for creating a term document management for a vector of open-text comments called ’comments’. modifications are made to the open text comments including the removal of punctuation and weighting using the TF-DF technique. The final matrix which is saved to an objects names ’x’ could The linked to a vector of outcomes ‘y’ and used to train and validate machine learning algorithms using the process described above listings 3 to 11.

Once created, documents in the TDM can be combined with a vector of outcomes using the cbind() function, as shown in Table 4, and processed in the same way as demonstrated in Fig. 7. Interested readers can explore the informative tm package documentation to learn more about term-document matrices [31].

## Results

When trained on a proportion of the dataset, the three algorithms were able to classify cell nuclei in the remainder of the dataset with high accuracy (.94 -.96), sensitivity (.97 -.99), and specificity (.85 -.94). Though each algorithm performed well individually, maximum accuracy (.96) and area under the curve (.97) was achieved using the SVM algorithm (see Table 3).

Model performance was marginally increased when the three algorithms were arranged into a voting ensemble, with an overall accuracy of.97, sensitivity of.99 and specificity of.95 (see the attached R Code for further details.).

## Discussion

Machine learning has the potential to transform the way that medicine works [32], however, increased enthusiasm has hitherto not been met by increased access to training materials aimed at the knowledge and skill sets of medical practitioners.

In this paper, we introduce basic ML concepts within a context which medical researchers and clinicians will find familiar and accessible. We demonstrate three commonly-used algorithms; a regularized general linear model, support vector machines (SVM), and an artificial neural network to classify tumour biopsies with high accuracy as either benign or malignant. Our results show that all algorithms can perform with high accuracy, sensitivity, and specificity despite substantial differences in the way that the algorithms work. The best-performing algorithm, the SVM, is very similar to the method demonstrated by Wolberg and Mangasarian who used different versions of the same dataset with fewer observations to achieve similar results [18, 33]. It is noteworthy that the LASSO-regularized linear regression also performed exceptionally well whilst preserving the ability to understand which features were guiding the predictions (see Table 5). In contrast, the archetypal ’black box’ of the heavily-parametrized neural network could not improve classification accuracy.

**Table 5:** Example features and coefficients from the GLM algorithm

Feature	Thickness	Cell Size	…	Epithelial Size	…	Bland Chromatin	…	Mitoses
Coefficient	0.49	0.11		0.02		0.48		0.36

In parallel to our analysis, we demonstrate techniques which can be applied with a commonly-used and open-source programming software (the R environment) which does not require prior experience with command-line computing. The presented code is designed to be re-usable and easily adaptable, so that readers may apply these techniques to their own datasets. With some modification, the same code may be used to develop linguistic classifiers or object recognition algorithms using open-text or image-based data respectively. Though the R environment now provides many options for advanced ML analyses, including deep learning, the framework of the code can be easily translated to other programming languages, such as Python, if desired. After working through examples in this paper we suggest that user apply their knowledge to problems within their own datasets. Doing so will elucidate specific issue which need to be overcome and will form a foundation for continued learning in this area. Further information can be from any number of excellent textbooks, websites, and online courses. Additional practice data sets can be obtained from the University of California Irvine Machine learning data sets repository which at the time of writing, includes an additional 334 datasets suitable for classification tasks, including 35 which contain open-text data [17].

Further, this paper acts to demystify ML and endow clinicians and researchers without a previous ML experience with the ability to critically evaluate these techniques. This is particularly important because without a clear understanding of the way in which algorithms are trained, medical practitioners are at risk of relying too heavily on these tools which might not always perform as expected. In their paper demonstrating a multi-surface pattern separation technique using a similar dataset, Wolberg and Mangasarian stress the importance of training algorithms on data which does not itself contain errors; their model was unable to achieve perfect performance as the sample in the dataset appeared to have been incorrectly extracted from an area beyond the tumour. The oft-told parable of the failure of the Google Flu Trends model offers an accessible example of the risks and consequences posed by a lack of understanding of ML models deployed ostensibly to improve health [34]. In short, the Google Flu Trends model was not generalizable over time as the Google Search data it was trained on was temporally sensitive. Looking to applications of ML beyond the medical field offers further insight into some risks that these algorithms might engender. For example, concerns have been raised about predictive policing algorithms and, in particular, the risk of entrenching certain prejudices in an algorithm which may be apparent in police practice. Though the evidence of whether predictive policing algorithms leads to biases in practice is unclear [35], it stands to reason that if biases exist in routine police work then models taught to recognize patterns in routinely collected data would have no means to exclude these biases when making predictions about future crime risk. Similar bias-based risks have been identified in some areas of medical practice and, if left unchecked, threaten the ethical use of data-driven automation in those areas [36]. An understanding of the way ML algorithms are trained is essential to minimize and mitigate the risks of entrenching biases in predictive algorithms in medicine.

## Conclusions

The approach which we have taken in this paper entails some notable strengths and weaknesses. We have chosen to use a publicly-available dataset which contains a relatively small number of inputs and cases. The data is arranged in such a way that will allow those trained in medical disciplines to easily draw parallels between familiar statistical and novel ML techniques. Additionally, the compact dataset enables short computational times on almost all modern computers. A caveat of this approach is that many of the nuances and complexities of ML analyses, such as sparsity or high dimensionality, are not well represented in the data. Despite the omission of these common features of a ML dataset, we are confident that users who have worked through the examples given here with the code provided in the appendix will be well-placed to further develop their skills working on more complex datasets using the scalable code framework which we provide. In addition, this data also usefully demonstrates an important principle of ML: more complex algorithms do not necessarily beget more useful predictions.

We look toward a future of medical research and practice greatly enhanced by the power of ML. In the provision of this paper, we hope that the enthusiasm for new and transformative ML techniques is tempered by a critical appreciation for the way in which they work and the risks that they could pose.

## Additional files

Additional file 1 Breast Cancer Wisconsin Dataset. Anonomised dataset used in this work. (CSV 24.9 kb)

Additional file 2 R Markdown Supplementary Material. R Code accompanying the work described in this paper and its output. (PDF 207 kb)

## Abbreviations

ANN: Artificial neural network
AUC: Area under the curve
FNA: Fine needle aspiration
GLM: Generalized linear model
IDE: Integrated developer environment
LASSO: Least absolute shrinkage and selection operator
ML: Machine learning
NLP: Natural language processing
RBF: Radial basis function
ROC: Received operating characteristics
SVM: Support vector machine
TD-IDF: Term document - inverse document frequency
TDM: Term document matrix
t-SNE: t-embedded stochastic neighbor embedding
UCI: University of California, Irvine
