# Supplementary material for: Machine learning in medicine: a practical introduction
Source: BMC Med Res Methodol. 2019 Mar 19;19:64. doi: 10.1186/s12874-019-0681-4 (PMC6425557; doi:10.1186/s12874-019-0681-4)
Supplement: Supplementary file 2 — R Markdown Supplementary Material. R Code accompanying the work described in this paper and its output. (PDF 207 kb) [file 12874_2019_681_MOESM2_ESM.pdf]

Machine learning in medicine : a practical introduction

Sidey-Gibbons JAM, Gibbons CJ

4/18/2018

R Markdown supplimentary material

This document accompanies the paper published

```
cancer = read.csv(paste0("http://archive.ics.uci.edu/ml/machine-learning-databases/",
    "breast-cancer-wisconsin/breast-cancer-wisconsin.data"), header = FALSE,
    stringsAsFactors = F)  # Load dataset from the UCI repository.

names(cancer) = c("ID", "thickness", "cell_size", "cell_shape", "adhesion",
    "epithelial_size", "bare_nuclei", "bland_cromatin", "normal_nucleoli", "mitoses",
    "class")  # Add names to the dataset.

cancer = as.data.frame(cancer)
cancer$bare_nuclei = replace(cancer$bare_nuclei, cancer$bare_nuclei == "?",
    NA)  # Recode missing values with NA.
cancer = na.omit(cancer)  # Remove rows with missing values.
cancer$class = (cancer$class/2) - 1  # Recode the class (outcome) variable to 1 and 2.

head(cancer)  # Show the first 6 rows of the dataset
```

```
##        ID thickness cell_size cell_shape adhesion epithelial_size
## 1 1000025         5         1          1        1               2
## 2 1002945         5         4          4        5               7
## 3 1015425         3         1          1        1               2
## 4 1016277         6         8          8        1               3
## 5 1017023         4         1          1        3               2
## 6 1017122         8        10         10        8               7
##   bare_nuclei bland_cromatin normal_nucleoli mitoses class
## 1           1              3               1       1     0
## 2          10              3               2       1     0
## 3           2              3               1       1     0
## 4           4              3               7       1     0
## 5           1              3               1       1     0
## 6          10              9               7       1     1
```

```
set.seed(80817)  # Set a random seed so that repeated analyses have the same outcome. Seeds are saved o
index = 1:nrow(cancer)  #Create an index vector with as many sequential variables as there are rows in
testindex = sample(index, trunc(length(index)/3))  #Take a sample of 33.3% of the variables from the in
testset = cancer[testindex, ]  #Create a test (validation) dataset with 33.3$ of the data.
trainset = cancer[-testindex, ]  #Create a trainig dataset with 66.6% of the data.

x_train = data.matrix(trainset[, 2:10])  # Take the features (x) from the training dataset.
y_train = as.numeric(trainset[, 11])  # Take the outcomes (y) from the training dataset.

x_test = data.matrix(testset[, 2:10])  # Take the features (x) from the testing/validation dataset.
y_test = as.numeric(testset[, 11])  # Take the outcomes (y) from the testing/validation dataset.

# You can use the dim() function to assess the dimension of each matrix
```

```r
# (e.g., dim(x_train))

# install.packages('glmnet',repos=getOption('repos')) Install latest verison
# of `glmnet`. Only necessary once.
```

```r
require(glmnet) # Load glmnet package into this R session.
glm_model = cv.glmnet(x_train, y_train, alpha=1, nfolds=10) # 10-fold cross validation of the LASSO-reg
lambda.min = glm_model$lambda.min # Save the lambda value which minimizes the error of the linear model
glm_coef = round(coef(glm_model,s= lambda.min),2) #Individual coefficients for variable included in the
```

```r
plot(glm_model) # Plots mean squared error against log(Lambda).
```

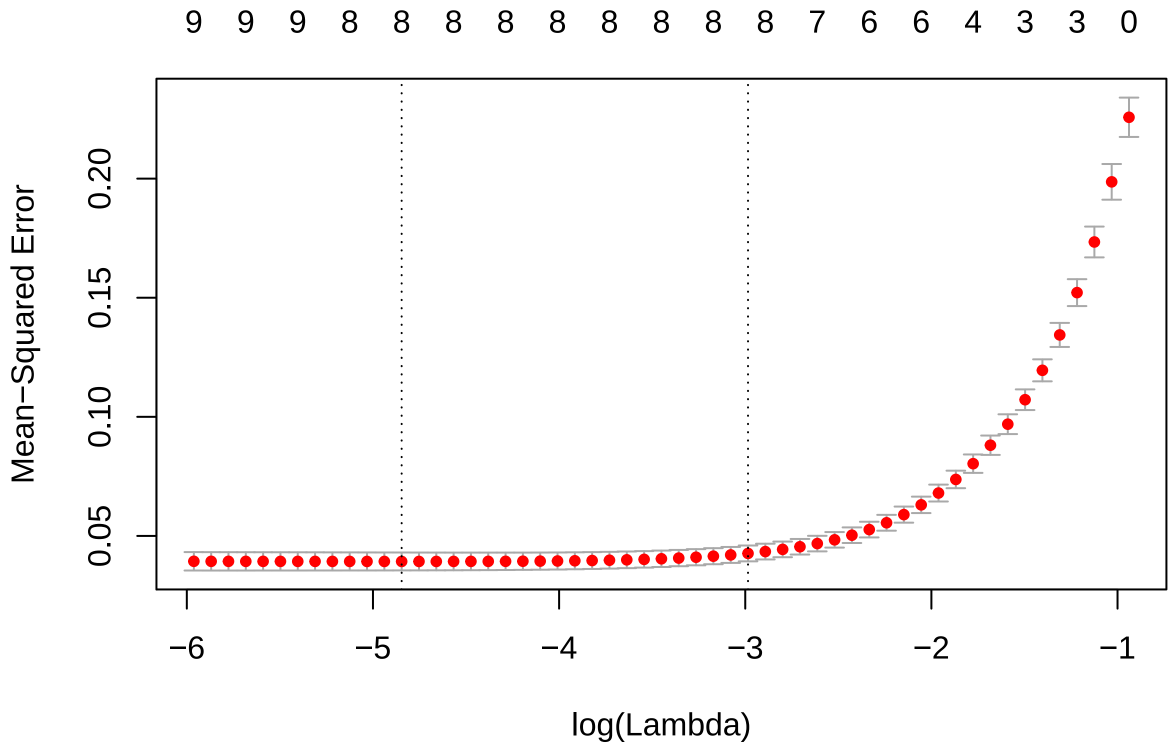


```r
plot(glmnet(x_train,y_train, family="gaussian", alpha=1),"lambda",label=T, main="") #Plots coefficient 
abline(v=log(lambda.min), lty=3) #Adds a vertical line to the plot of line 34 at the minimum level of l
```

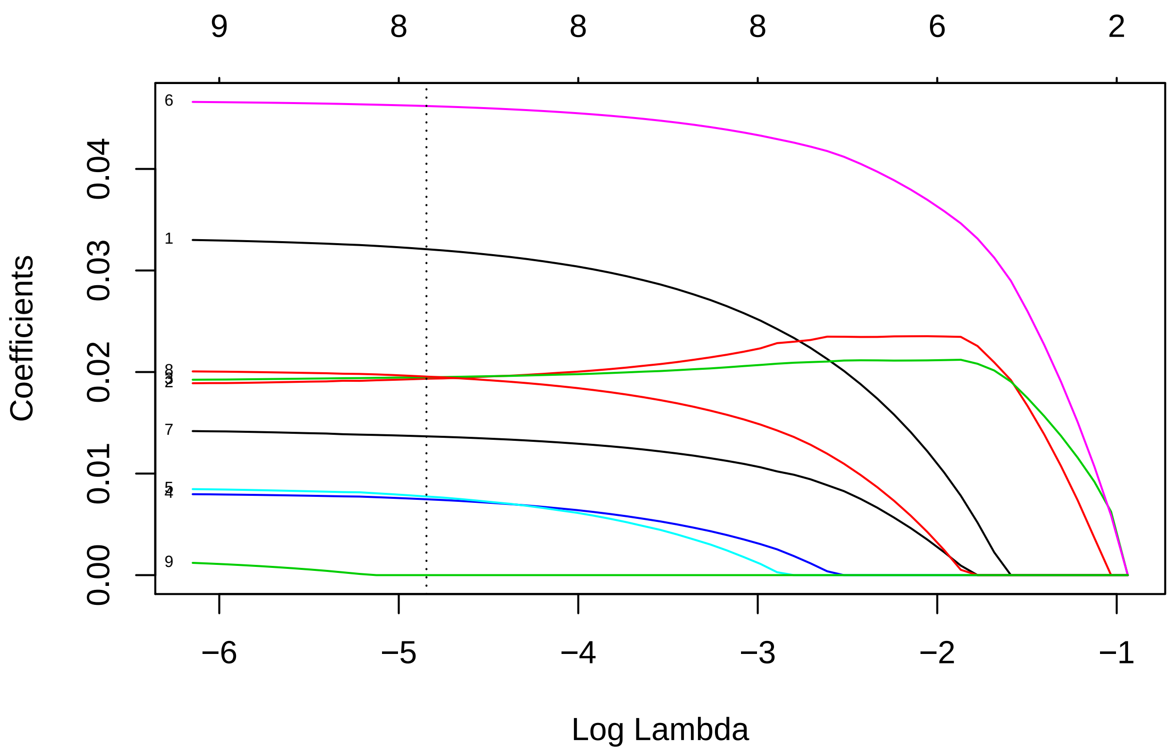


```r
#install.packages("e1071") # Install latest verison of `e1071`. Only necessary once.
require(e1071)  # Load e1071 package into this R session.
```

```
## Loading required package: e1071
```

```r
svm_model = svm(x_train, y_train, cost = 1, gamma = c(1/(ncol(x_train)-1)), kernel="radial", cross=10)
```

```
## Warning in cret$cresults * scale.factor: Recycling array of length 1 in vector-array arithmetic is d
##   Use c() or as.vector() instead.
```

```r
#install.packages("nnet") # Install latest verison of `nnet`. Only necessary once.
require(nnet) # Load e1071 package into this R session.
```

```
## Loading required package: nnet
```

```r
nnet_model = nnet(x_train, y_train, size=5) #Fit a single-layer neural network to the data with 5 units
```

```
## # weights:  56
## initial  value 112.078697
## iter  10 value 45.002542
## final  value 40.999975
## converged
```

```r
glm_pred = round(predict(glm_model, x_test, type="response"),0) # Create a vector of predicitons made f
svm_pred = round(predict(svm_model, x_test, type="response"),0) #Prediction vector for the SVM.
nnet_pred = round(predict(nnet_model, x_test, type="raw"),0) #Prediction vector for the neural network.

predictions = data.frame(glm_pred,svm_pred,nnet_pred) # Collate the three prediction vectors into a dat
names(predictions) = c("glm","svm","nnet") #Name the columns of the dataframe.

predictions$sum = rowSums(predictions)  # Create a new column in the predictions dataset of the sum of
algorithms_n = 3 #Insert how many algorithms you have in your predictions data frame. In this case ther
predictions$ensemble_votes = round(predictions$sum/algorithms_n) #Create a new column containing the vo
```

```r
print(predictions$ensemble_votes[1:30]) # Print the first 30 objects in the vector of predictions from
```

```
##  [1] 0 0 0 0 0 1 0 0 0 1 0 1 0 0 0 0 0 0 1 0 1 0 1 0 0 1 0 0 0 1
```

```r
#install.packages("caret") # Install the `caret` package. Only necessary once.
require(caret) # Load the caret package into this R session.
```

```
## Loading required package: caret

## Loading required package: lattice

## Loading required package: ggplot2
```

```r
confusionMatrix(as.factor(glm_pred),as.factor(y_test))# Create a confusion matrix for the LASSO linear
```

```
## Confusion Matrix and Statistics
##
##           Reference
## Prediction   0   1
##          0 143   7
##          1   2  75
##
##                Accuracy : 0.9604
##                  95% CI : (0.9261, 0.9817)
##     No Information Rate : 0.6388
##     P-Value [Acc > NIR] : <2e-16
##
##                   Kappa : 0.9129
##  Mcnemar's Test P-Value : 0.1824
##
##             Sensitivity : 0.9862
##             Specificity : 0.9146
##          Pos Pred Value : 0.9533
##          Neg Pred Value : 0.9740
##              Prevalence : 0.6388
##          Detection Rate : 0.6300
##    Detection Prevalence : 0.6608
##       Balanced Accuracy : 0.9504
##
##        'Positive' Class : 0
##
```

```r
confusionMatrix(as.factor(svm_pred),as.factor(y_test)) # Create a confusion matrix for the SVM.
```

```
## Confusion Matrix and Statistics
##
##           Reference
## Prediction   0   1
##          0 143   2
##          1   2  80
##
##                Accuracy : 0.9824
##                  95% CI : (0.9555, 0.9952)
##     No Information Rate : 0.6388
##     P-Value [Acc > NIR] : <2e-16
##
##                   Kappa : 0.9618
```

```
##  Mcnemar's Test P-Value : 1
##
##             Sensitivity : 0.9862
##             Specificity : 0.9756
##          Pos Pred Value : 0.9862
##          Neg Pred Value : 0.9756
##              Prevalence : 0.6388
##          Detection Rate : 0.6300
##    Detection Prevalence : 0.6388
##       Balanced Accuracy : 0.9809
##
##        'Positive' Class : 0
##
```

```
confusionMatrix(as.factor(nnet_pred),as.factor(y_test)) # Create a confusion matrix for the neural netw
```

```
## Confusion Matrix and Statistics
##
##           Reference
## Prediction   0   1
##          0 130   8
##          1  15  74
##
##                Accuracy : 0.8987
##                  95% CI : (0.8519, 0.9347)
##     No Information Rate : 0.6388
##     P-Value [Acc > NIR] : <2e-16
##
##                   Kappa : 0.7844
##  Mcnemar's Test P-Value : 0.2109
##
##             Sensitivity : 0.8966
##             Specificity : 0.9024
##          Pos Pred Value : 0.9420
##          Neg Pred Value : 0.8315
##              Prevalence : 0.6388
##          Detection Rate : 0.5727
##    Detection Prevalence : 0.6079
##       Balanced Accuracy : 0.8995
##
##        'Positive' Class : 0
##
```

```
confusionMatrix(as.factor(predictions$ensemble_votes),as.factor(y_test))  # Create a confusion matrix f
```

```
## Confusion Matrix and Statistics
##
##           Reference
## Prediction   0   1
##          0 143   4
##          1   2  78
##
##                Accuracy : 0.9736
##                  95% CI : (0.9434, 0.9902)
##     No Information Rate : 0.6388
```

```
##     P-Value [Acc > NIR] : <2e-16
##
##                   Kappa : 0.9424
##  Mcnemar's Test P-Value : 0.6831
##
##             Sensitivity : 0.9862
##             Specificity : 0.9512
##          Pos Pred Value : 0.9728
##          Neg Pred Value : 0.9750
##              Prevalence : 0.6388
##          Detection Rate : 0.6300
##    Detection Prevalence : 0.6476
##       Balanced Accuracy : 0.9687
##
##        'Positive' Class : 0
##
```

```
#install.packages("pROC") # Install the `pROC` package. Only necessary once.
require(pROC) # Load the caret package into this R session.
```

```
## Loading required package: pROC
```

```
## Type 'citation("pROC")' for a citation.
```

```
##
## Attaching package: 'pROC'
```

```
## The following object is masked from 'package:glmnet':
##
##     auc
```

```
## The following objects are masked from 'package:stats':
##
##     cov, smooth, var
```

```
roc_glm = roc(as.vector(y_test),as.vector(glm_pred)) #Conduct the ROC analyses
roc_svm = roc(as.vector(y_test), as.vector(svm_pred))
roc_nnet = roc(as.vector(y_test), as.vector(nnet_pred))

plot.roc(roc_glm, ylim=c(0,1), xlim=c(1,0)) #Plot the ROC curves
lines(roc_glm, col="blue")
lines(roc_nnet, col="green")
lines(roc_svm, col="red")
legend("bottomright", legend=c("General Linear Model", "Support Vector Machine", "Neural
                               Net"), col=c("blue","red","green"), lwd=2)
```

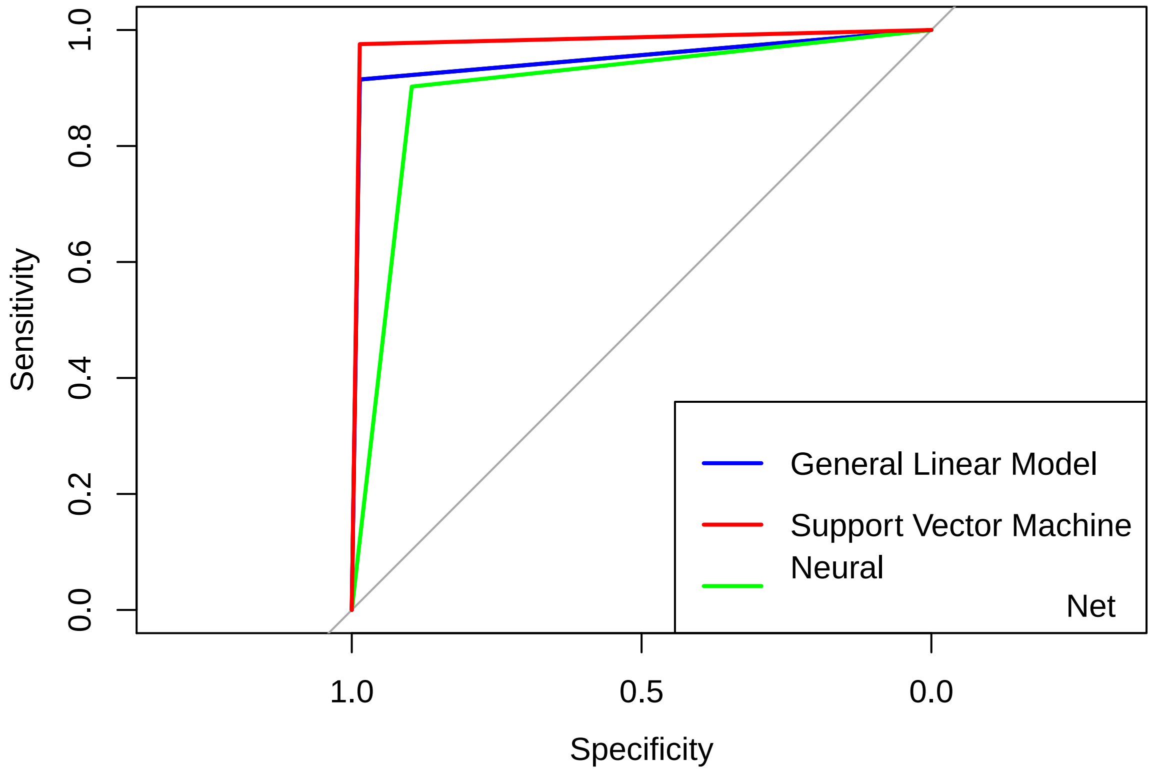


```
auc_glm = auc(roc_glm)#Calculate the area under the ROC curve
auc_svm = auc(roc_svm)#Calculate the area under the ROC curve
auc_nnet = auc(roc_nnet)#Calculate the area under the ROC curve
```

```
# The code below sets the values for the features to be evaluated by the trained and validated model.
thickness = 8
cell_size = 7
cell_shape = 8
adhesion = 5
epithelial_size = 5
bare_nuclei = 7
bland_cromatin = 9
normal_nucleoli = 8
mitoses = 10

new_data = c(thickness,cell_size,cell_shape,adhesion,
                epithelial_size,bare_nuclei,bland_cromatin,normal_nucleoli ,mitoses) #Comine the data t

new_pred_glm = predict(glm_model ,data.matrix(t(new_data))
                         ,type="response") #Apply the new data to the validated model
new_pred_svm = predict(svm_model ,data.matrix(t(new_data))
                         ,type="response")
new_pred_nnet = predict(nnet_model ,data.matrix(t(new_data)),type="raw")

print(new_pred_glm) #Print the prediction for the new data from the glm.
```

```
##              1
## [1,] 0.9139385
```

```r
print(new_pred_svm) #Print the prediction for the new data from the svm.
```

```
##         1
## 0.9803988
```

```r
print(new_pred_nnet) #Print the prediction for the new data from the nnet.
```

```
##           [,1]
## [1,] 0.9999967
```
